# Beyond HIV prevention: everyday life priorities and demand for PrEP among Ugandan HIV serodiscordant couples

**DOI:** 10.1002/jia2.25225

**Published:** 2019-01-18

**Authors:** Edith Nakku‐Joloba, Emily E Pisarski, Monique A Wyatt, Timothy R Muwonge, Stephen Asiimwe, Connie L Celum, Jared M Baeten, Elly T Katabira, Norma C Ware

**Affiliations:** ^1^ Department of Epidemiology and Biostatistics Makerere University College of Health Sciences Kampala Uganda; ^2^ STD Clinic/Ward 12 Mulago Hospital Kampala Uganda; ^3^ Department of Global Health and Social Medicine Harvard Medical School Boston MA USA; ^4^ Harvard Global Cambridge MA USA; ^5^ Infectious Diseases Institute Makerere University Kampala Uganda; ^6^ Kabwohe Clinical Research Centre Kabwohe Uganda; ^7^ Departments of Global Health, Medicine, and Epidemiology School of Medicine and School of Public Health University of Washington Seattle WA USA; ^8^ Department of Medicine Makerere University Kampala Uganda; ^9^ Department of Medicine Brigham & Women's Hospital Boston MA USA

**Keywords:** prevention, PrEP, HIV, serodiscordant couples, East Africa, demand creation

## Abstract

**Introduction:**

Pre‐exposure prophylaxis (PrEP) to prevent HIV infection is being rolled out in Africa. The uptake of PrEP to date has varied across populations and locations. We seek to understand the drivers of demand for PrEP through analysis of qualitative data collected in conjunction with a PrEP demonstration project involving East African HIV serodiscordant couples. Our goal was to inform demand creation by understanding what PrEP means – beyond HIV prevention – for the lives of users.

**Methods:**

The Partners Demonstration Project evaluated an integrated strategy of PrEP and antiretroviral therapy (ART) delivery in which time‐limited PrEP served as a “bridge” to long‐term ART. Uninfected partners in HIV serodiscordant couples were offered PrEP at baseline and encouraged to discontinue once infected partners had taken ART for six months. We conducted 274 open‐ended interviews with 93 couples at two Ugandan research sites. Interviews took place one month after enrolment and at later points in the follow‐up period. Topics included are as follows: (1) discovery of serodiscordance; (2) decisions to accept/decline PrEP and/or ART; (3) PrEP and ART initiation; (4) experiences of using PrEP and ART; (5) PrEP discontinuation; (6) impact of PrEP and ART on the partnered relationship. Interviews were audio‐recorded and transcribed. We used an inductive, content analytic approach to characterize meanings of PrEP stemming from its effectiveness for HIV prevention. Relevant content was represented as descriptive categories.

**Results:**

Discovery of HIV serodiscordance resulted in fear of HIV transmission for couples, which led to loss of sexual intimacy in committed relationships, and to abandonment of plans for children. As a result, partners became alienated from each other. PrEP countered the threat to the relationship by reducing fear and reinstating hopes of having children together. Condom use worked against the re‐establishment of intimacy and closeness. By increasing couples’ sense of protection against HIV infection and raising the prospect of a return to “live sex” (sex without condoms), PrEP was perceived by couples as solving the problem of serodiscordance and preserving committed relationships.

**Conclusions:**

The most effective demand creation strategies for PrEP may be those that address the everyday life priorities of potential users in addition to HIV prevention.

**Clinical Trial Number:**

NCT02775929

## 1 Introduction

Pre‐exposure prophylaxis (PrEP) has proven highly effective in preventing HIV infection when taken regularly 1, 2, 3. Moreover, PrEP can be delivered safely, with high uptake and adherence by users 4. A number of sub‐Saharan African countries have acted on these findings to begin making PrEP publicly available. South Africa launched an initiative to provide PrEP to female sex workers in early 2016 5. The Strategic Plan for 2017 to 2022 expands access to include men who have sex with men (MSM), injection drug users and youth 6. In May 2017, the Government of Kenya launched an initiative to provide PrEP as part of combination HIV prevention. Currently, PrEP is being made available to serodiscordant couples, sex workers, adolescent girls/young women and other populations at high risk for HIV infection in a variety of delivery settings 7, 8. The Ugandan government is offering PrEP to key populations at a number of accredited health facilities around the country 9. Several other countries in East, West and southern Africa have PrEP implementation projects 10.

Information currently available on PrEP uptake in Africa suggests a varied initial response. As of July 2018, the number of PrEP initiations in Kenya stood at two‐thirds of national targets, in Zimbabwe at 50% of targets, and in South Africa at 41% of targets 11. In South Africa's step‐wise population‐based approach to PrEP scale‐up, 13% of sex workers offered PrEP in the initial stage (starting June 2016) initiated, in contrast to 54% of MSM in the second stage (starting April 2017), and 6% of university students in the third stage (October 2017) 12. Reports of challenges encountered and lessons learned from implementation initiatives are now appearing.

Retention is developing into a major challenge, with individuals initiating or expressing interest in PrEP choosing not continue due to medication side effects, fear of stigma, negative attitudes from clinical staff and other considerations 13, 14. Underestimation of risk may impede uptake, insofar as at‐risk individuals perceive their HIV risk to be low 15. Lack of awareness continues to be cited as a barrier to PrEP uptake 16.

In sum, limited initial demand is emerging as an important influence on the uptake of PrEP in Africa. Activities aimed at generating interest in new products and technologies by linking them to the priorities of prospective users are known as *demand creation* 17. Creating demand for PrEP by linking it to user priorities requires understanding what those priorities are. User priorities may extend beyond HIV prevention and may differ across population groups. This paper addresses PrEP demand creation for East African serodiscordant couples by examining their life priorities. We approach this using qualitative data to describe what PrEP as an effective HIV prevention tool meant to a group of Ugandan serodiscordant couples using it as part of the Partners Demonstration Project.

## 2 Methods

### 2.1 Study design and setting

This was a qualitative study carried out in conjunction with the Partners Demonstration Project. The Partners Demonstration Project (Clinicaltrials.gov NCT02775929) was an open‐label evaluation of integrated delivery of PrEP and antiretroviral therapy (ART) for higher‐risk HIV serodiscordant couples in Kenya and Uganda 4, 18. A validated, empiric risk scoring tool was utilized to recruit couples at higher risk of HIV transmission, who would benefit from the integrated strategy of PrEP and ART delivery 19.

The integrated delivery strategy offered time‐limited PrEP to uninfected partners in serodiscordant couples as a “bridge” to long‐term ART in infected partners. Uninfected partners were offered PrEP at baseline and encouraged to discontinue once infected partners had taken ART for six months. Counsellors also encouraged condom use to prevent sexually transmitted infections and unintended pregnancies 20. At the outset of the project, only individuals with CD4 counts ≤350 were eligible for ART initiation. Ugandan national treatment guidelines were revised in 2016 to include any HIV‐infected person in a serodiscordant relationship 9.

### 2.2 Sampling and recruitment

Purposeful sampling was used to identify participants in the qualitative study 21. We sought to purposefully sample couples with varying experiences of PrEP and ART. We included couples in which uninfected partners accepted and declined PrEP at enrolment, and couples in which the infected partner was eligible and ineligible for ART (e.g. based on CD4 < 350 prior to 2016). Couples in these categories were referred to the qualitative study by project staff. Research assistants approached these couples during follow‐up visits to describe the qualitative study and invite participation. Ninety‐three couples accepted and were enrolled.

### 2.3 Data collection

Qualitative data collection for this study took place at the Partners Demonstration Project's two Ugandan sites: the Infectious Diseases Institute – Kasangati, in Kampala; and Kabwohe Clinical Research Center, in the rural southwest. Multiple open‐ended interviews were carried out with couples participating in the qualitative study.

Interviews took place approximately one month after enrolment in the Partners Demonstration Project, and at later points in the follow‐up period. Examples of interview topics included: (1) discovery of serodiscordance; (2) decisions to accept/decline PrEP and/or ART; (3) experiences of PrEP and ART initiation; (4) experiences of using PrEP and ART; (5) PrEP discontinuation; and (6) the impact of PrEP and ART on the partnered relationship. Partners took part in initial interviews together, to allow insight into relationship dynamics. Subsequent interviews were a mix of individual and joint sessions, depending on the topics to be discussed. Two hundred and seventy‐four interviews were completed. One hundred and forty‐eight were interviews with both members of the couple; 126 were individual interviews.

Interviews were conducted by trained Ugandan research assistants in local languages (Luganda, Runyankore), using interview guides. Each interview type had a different guide, tailored to the experience being investigated. In the initial joint interviews, the interviewer took notes on relationship dynamics, guided by a predesignated list of relationship characteristics.

Interviews were conducted in private settings in locations selected by interviewees. Participants provided written consent for interviews, which were audio‐recorded and lasted about an hour. Audio‐recordings were transcribed into English by the research assistants. Transcripts were reviewed for content and technique in weekly feedback phone calls and emails with a supervisor to ensure data quality. Besides the transcripts, the research assistants prepared “debriefs” summarizing interview content. Interview data were collected from November 2013 through December 2016.

### 2.4 Data analysis

An inductive, content analytic approach was used to analyse the qualitative data 22. Transcripts were initially reviewed as they were produced, to provide an overall sense of the content. A coding scheme was developed from this process; the dataset was coded using Atlas.ti qualitative data management software. For the analysis reported here, the primary and the senior author reviewed coded data that spoke to meanings stemming from the effectiveness of PrEP for preventing HIV transmission. Where indicated by the review of coded data, selected complete transcripts were re‐read.

This process led to the preliminary specification of concepts addressing the research question. Coded and transcript data were then repeatedly reviewed to assign examples to the preliminary concepts. The addition of examples served to refine and elaborate the concepts, transforming them into descriptive categories. Statements summarizing the content of each category were added, along with evidence in the form of illustrative quotes from interview transcripts. Finally, the categories were linked to “tell the story” of the meanings of PrEP that emerged from the analysis.

### 2.5 Ethics approval

Ethical approvals were obtained from the Committee on Human Studies, Harvard Medical School, Boston MA; the National HIV/AIDS Research Committee of the Ugandan National Council for Science and Technology, Kampala; and the University of Washington Institutional Review Board, University of Washington, Seattle, WA.

## 3 Results

### 3.1 Participant characteristics

Couples eligible for the Partners Demonstration Project were ≥18 years of age, sexually active and reported intending to remain together.

HIV‐infected and uninfected partners in the qualitative study were in their early thirties. Approximately half (46%) of uninfected partners were female. Median time since discovering serodiscordant status was two months at baseline (Range: 1 to 12). Median time living together was three years (Range: 1 to 9). Almost all couples (N = 91, 98%) reported being married to each other. Fifty‐three percent of couples (N = 49) had children together at baseline.

Among qualitative study participants, 88% (N = 82) of uninfected partners initiated PrEP at Partners Demonstration Project enrolment. PrEP initiation increased to 92% (N = 86) during the follow‐up period. Sixty‐six percent (N = 61) of infected partners among qualitative study participants were eligible for ART at enrolment. Sixty‐seven percent (N = 40) of eligible individuals initiated ART at enrolment; all initiated ART during the follow‐up period. Twenty‐one (23%) couples in the qualitative sample reported ending their relationship after enrolling in the Partners Demonstration Project (Table 1).

**Table 1:** Characteristics of couples participating in the qualitative study (N = 93 couples)

	Median (IQR) or N (%) Total
Characteristics, HIV uninfected partner	
Age, years	31 (26 to 37)
Female Sex	43 (46%)
Initiated PrEP at enrolment	82 (88%)
Initiated PrEP at enrolment or during follow‐up period	86 (92%)
Characteristics, infected partner	
Age, years	31 (25 to 37)
ART eligible, project enrolment	61 (66%)
Initiated ART within 15 days of enrolment, among ART eligible individuals (N = 60)	40 (67%)
Initiated ART during follow‐up period (N = 91)^a^	91 (100%)
Characteristics, couple	
Time since learning of HIV serodiscordance, months	2 (1 to 12)
Living together, years	3 (1 to 9)
Married to each other	91 (98%)
Children together	49 (53%)
Children together, median	1 (0 to 2)
Ended relationship during the follow‐up period	21 (23%)

a ART initiation data are not available for two participants.

### 3.2 Meanings of PrEP

#### 3.2.1 Discovery of HIV serodiscordance threatened partnered relationships

Discovery of HIV serodiscordance resulted in fear of HIV transmission for couples. This in turn led to the loss of sexual interest and sexual intimacy between partners, distancing them from each other. Serodiscordance also suggested infidelity, creating anger and distrust, and exacerbating alienation.

Couples responded to the discovery of serodiscordance in different ways. Some took steps to reduce transmission risk – by decreasing frequency of sexual intercourse, abstaining from sex altogether, starting to use condoms or making efforts to use them more frequently. These risk reduction steps further eroded intimacy, creating additional distance between partners (Table 2, A, 1).

**Table 2:** Data excerpts illustrating content of descriptive categories

Summary Statement	Elaboration	Data excerpts
A. Discovery of serodiscordance threatened partnered relationships.	1. Steps to reduce transmission risk erode intimacy and create distance between partners	“F: …He so much avoids being near me. He is not close to me. He is not so free with me. He is no longer like before we tested. I am not happy because he avoids having sex with me. The love was too much before but it has currently reduced. I show him that I love him but he does not. Okay he loves me but he is not so much close to me. I: *What do you mean by him not being so close to you?* F: For instance we can only have sex once in a month. Sometimes we can have sex once in two months and he seems like he does not want to have children and he does not stay with me most of the time.” HIV‐uninfected Female, Age 20
	2. Postponing or abandoning plans for having children	“That's why we decided that we should not produce more children – to avoid getting HIV. We may decide to have live sex to conceive and I get HIV, so we decided we won't have more children and take care of the ones we already have.” HIV‐uninfected Female, Age 26
	3. Considering separation	“I did not want [my wife] to leave; only that we were no longer doing things that we used to do before. …I told her that we shall be having sex once in a week or once in two weeks, unlike before when we could have sex every day or two days. I told her that in order to avoid risks. I think that is the reason why she decided to leave.” HIV‐uninfected Male, Age 34
B. PrEP countered the threat to the relationship by reducing fear, and reinstating hopes and plans for family building.	1. Reduced fear of infection through added protection from PrEP re‐awakens sexual desire, bringing partners closer together	“…the desire to have sex. That desire can reduce when you are with a person that you do not trust very well, yes. But what encourages is when you know that you have medicine (PrEP) that you can take such that you can have sex with this person without getting infected, that is, when you also have a condom. So there you get the desire to have sex.” HIV‐infected Male, Age 54
	2. PrEP (and ART) restore plans for having children	*“*I: *How many do children intend to have?* F: Like two more… M: The good thing is we were told we can do it without her infecting me with the virus. I: *What were you told exactly?* M: Now that she is on ART and I am on PrEP, her chances of infecting me are minimal. So when the time comes to have another child, we shall not use condoms.” HIV‐infected Female, Age 21; HIV‐uninfected Male, Age 28
C. Couples struggled to combine PrEP with condom use, as they experienced condoms as working against the re‐establishment of intimacy and closeness.	1. Condom use in a committed relationship connotes sex with outside partners	“You see people who use condoms normally are those who are sleeping with other partners outside their marriage. That's why I find it a bit awkward to be using a condom with my own wife. It appears as if you are sleeping with another woman and not someone you are committed to as your wife.” HIV‐uninfected Male, Age 27
	2. Some couples are able to adjust to condom use	“When we started using condoms, I used to feel nothing. It was like that I had no sex. Even my husband – the condom disturbed him because we could start having sex but in the middle the man lose appetite for sex. But nowadays things are going well, it's like we got used to it.” HIV‐infected Female, Age 35
	3. Couples desire a return to “live sex”	“We want to hear…that someone who takes PrEP is safe and can have live sex. What is the importance of [partner] taking PrEP if we are still using condoms?” HIV‐uninfected Female, Age 30
	4. Couples see PrEP as a way of avoiding HIV transmission while remaining in the serodiscordant relationship	“That is why we got used to being serodiscordant quickly; because we love each other. And now, the medicine we both take allows us to continue our relationship.” HIV‐uninfected Female, Age 27 [PrEP]‐ “helped us and this is how it helped us. You do not separate from your wife, you remain together. [Before PrEP] I would fear, ‘this happened to my wife so what should we do? Maybe we should separate.’ But now we remain together and continue moving on.” HIV‐uninfected Male, Age 42

Couples also responded to HIV serodiscordance by abandoning or postponing plans for having children. Loss of family building as a shared goal meant losing a reason to be together. Reasons for changing plans centred on fear of not being able to support children into adulthood if the HIV‐infected partner died or became incapacitated, or if the HIV‐negative partner became infected (Table 2, A, 2).

The bond between them weakened, some couples considered separation. Their relationships were not able to withstand the cumulative stress of serodiscordance on top of economic and other challenges. These couples saw separation as the most reliable means of ensuring the uninfected partner remained free of HIV, or found coping with risk reduction measures unacceptably burdensome (Table 2, B, 1).

#### 3.2.2 PrEP countered threats to relationships by reducing fear, and reinstating hopes and plans for family building

PrEP countered threats to relationships by reducing fear and reinstating hopes and plans for family building.

As indicated above, couples receiving PrEP and ART as part of the integrated strategy were encouraged to combine condoms with antiretrovirals for maximum protection against unwanted pregnancies as well as sexually transmitted infections 20. While they understood this, couples also tended to continue to think of condoms as a means of preventing HIV. They described “feeling safer” as a result of adding PrEP as an HIV prevention method to the method(s) they were already using. Couples often characterized PrEP as “back‐up” to condoms, protecting them if condoms broke or failed for another reason**.** Insofar as multiple protection methods reduced fear of HIV, the threat to the relationship also decreased, and alienated partners once again grew closer to each other. A reawakening of sexual desire was often part of this new closeness (Table 2, B, 2).

Also, couples came to accept PrEP as a safe and simple alternative to artificial insemination for safe conception. With increasing PrEP experience, viral suppression in the HIV‐infected partner, and support from project staff, couples learned to time sex without a condom to coincide with peak fertility periods—conceiving, as one woman put it, “like human beings do.” In this way, the hopes of HIV serodiscordant couples to have children together were restored through PrEP; the restoration of hope provided a reason for remaining in the relationship (Table 2, C, 2).

#### 3.2.3 Couples struggled to combine PrEP with condom use, as they experienced condoms as working against the re‐establishment of intimacy and closeness made possible through PrEP

Qualitative study couples described working hard to follow the advice of staff and integrate condoms and PrEP in their sex lives. But they struggled, since they also experienced condoms as working against newly re‐established intimacy and closeness.

Couples complained that condoms interfered with sexual pleasure and performance, causing them to once again lose interest in sexual activity. They found condoms to be especially problematic in a committed relationship, in that they suggest sex with outside partners. The introduction of condoms into a committed relationship by one or another partner could be offensive, connoting a lack of trust and suspicion of unfaithfulness (Table 2, C, 1).

Some couples adopted a compromise position between competing desires for HIV prevention and sexual satisfaction – adhering to condoms whenever possible, while also periodically indulging the urge not to use them. Others found themselves able to adjust to condom use over time (Table 2, C, 2).

Couples struggling with condom use found comfort in the hope that PrEP would eventually eliminate the need for them. They looked forward to a time when increasing recognition of the effectiveness of PrEP would make barrier methods for HIV prevention unnecessary, allowing for what they termed “live sex” (Table 2, C, 3).

A return to “live sex” made possible through PrEP promised increased intimacy and closeness. Live sex was considered better sex, increasing sexual pleasure in the relationship. Moreover, an uninfected partner remaining free of HIV as a result of PrEP meant that partner would be available to provide for the HIV‐infected partner, should his or her health deteriorate. Some uninfected partners saw PrEP use as a way of sharing the burden of HIV prevention in the relationship. Many qualitative study participants spoke of PrEP as a solution to the “problem” of serodiscordance – a way of avoiding HIV transmission while remaining in the relationship (Table 2, C, 4).

## 4 Discussion

This qualitative analysis sought to characterize meanings of PrEP beyond HIV prevention among Ugandan serodiscordant couples participating in the Partners Demonstration Project. Our findings reveal the primary meaning of PrEP for these couples to be its role in reversing the alienation and discord introduced into committed relationships by the discovery of serodiscordance. The roots of this alienation are complex, beginning with fear of HIV infection, and expanding to include larger life disappointments, such as feeling unable to fulfil personal goals and cultural expectations for family building, and experiencing the erosion of intimacy and trust that comes with condom use. For couples participating in this qualitative study, PrEP reversed alienation by reducing fear, making safe conception possible without recourse to “artificial” methods, and awakening the hope for increased satisfaction and closeness through a return to “live sex.” These effects combined to strengthen and restore threatened relationships.

HIV serodiscordant couples participating in this qualitative study reported PrEP strengthened relationships by reducing fear of HIV transmission and increasing sexual intimacy. These themes have also been reported in other couples‐focused analyses 23, 24, 25, 26, 27, 28, and among MSM PrEP users, most of whom are not in a known HIV serodiscordant relationship 29, 30. In this analysis, we draw out the larger significance of couples’ views, to consider their implications for future PrEP demand creation initiatives.

There has been considerable debate over whether access to PrEP and ART would result in the abandonment of condoms for prevention of HIV and other sexually transmitted infections 31, 32, 33, 34. A growing body of research suggests this is not necessarily the case 23, 30, 35, 36, 37. When condoms are not used, there may be several reasons – pursuit of pleasure, desire to be free of barriers and hope for children. In some circumstances, intimacy and relationship strengthening may take precedence over prevention of infection 38, 39.

The desire for “live sex” – sex without condoms – was strong in this sample of serodiscordant couples, and the hope that PrEP might open the door to “live sex” in the relationship was seen as an important benefit. In the meantime, couples tried hard to follow the recommendation of staff to use condoms consistently. Some couples acknowledged engaging in sex without condoms, but characterized this as the exception in an overall pattern of condom use, stemming from the desire to conceive a child, or “treat” themselves to a more pleasurable sexual experience.

PrEP served as a “bridge” to ART in the integrated strategy. Uninfected partners in serodiscordant couples took PrEP until their infected partners had used ART for six months. Whereas overall, PrEP users “felt safer” as a result of taking the medication, they were less confident of the protection afforded by their partner's ART. The concept of “treatment as prevention” was understood, but not widely accepted by PrEP users participating in this qualitative study. As a result, ART did not have the same meaning for couples, or exert the same impact on the serodiscordant relationship 40.

The question arises as to whether PrEP may have different meanings for male and female users. Insofar as women may face greater difficulty in negotiating condom use, they may disproportionately benefit from and appreciate a method of HIV prevention that allows them more agency and control. Men, in contrast, may interpret clinic visits and daily medication as part of “women's domains,” and feel more burdened by PrEP use as a result 41. Characterization of gender differences in meanings of PrEP for serodiscordant couples was not a focus of the analysis reported here.

Our results contribute to the emerging critique of PrEP demand creation strategies that are focused narrowly on risk reduction 38, 42, 43, 44. Reducing HIV risk is an important argument to make for PrEP uptake, but adding messaging that reflects what users describe as important additional benefits may increase interest and demand for PrEP.

This study and others suggest that serodiscordant couples see HIV transmission risk reduction through PrEP as the means to larger and more inherently appealing ends – freedom from fear during sex, reinstatement of plans for children, a return to “live sex,” and ultimately, the preservation of a committed relationship. The importance of relief from fear as a benefit of PrEP use has also been noted in qualitative research with male PrEP users participating in iPrEx 30. The cultural as well as personal significance of producing children, and the stigma of infertility have been described for Ugandan serodiscordant couples 45. Options for sex without condoms might be included in counselling and education sessions with couples considering PrEP use. Such sessions would make clear the relative roles of PrEP and condoms in preventing HIV and other sexually transmitted infections, while defining decisions about condom use as the choice and responsibility of couples themselves. Couples’ own characterizations of the role of PrEP in preserving relationships could be shared in descriptive materials. Messaging that maps PrEP onto the everyday life priorities of potential users in an “optimistic” way may ultimately prove more effective for demand creation than framing messaging content in terms of HIV prevention alone.

Meaningful efforts to inform PrEP demand creation will be grounded in a recognition of the varying approaches to implementation being adopted across Africa. For example, in Uganda, scale‐up efforts were initially led by academic researchers and advocates, who lobbied the government for access to PrEP through the public health system. Guidelines for providing PrEP were developed by a multi‐stakeholder working group and approved in July 2017. The current (2017 to 2018) programme for distribution is spearheaded by the AIDS Control Programme (ACP) of the Ministry of Health, which has worked to create demand through training for healthcare providers, increasing capacity for HIV testing in clinics, and instituting a clinic accreditation programme. Scale‐up is taking place in public health clinics across the country; the number of clinics distributing PrEP is being increased each year. In October 2018, reported PrEP initiations total 9000 to 9500 11.

In Kenya, a public messaging campaign directly targeting prospective PrEP users plays a prominent role in scale‐up. The adoption of positive rather than “fear‐based” messaging is a core principle of the campaign. Positive messaging highlights PrEP's contribution to happiness and wellbeing, rather than its role in reducing the risk of acquiring a potentially life‐threatening disease. Messaging materials are distributed widely outside as well as inside the healthcare system 46, 47.

Fitting PrEP demand creation strategies to the life priorities and meanings of PrEP for users is a principle that spans specific population groups. However, it requires understanding the life experiences and goals of group members, from their own points of view. Research identifying the nurturing of intimate relationships as a priority for couples – heterosexual and MSM – is a first step. Similar inquiries focusing on other key populations may help to effectively address suboptimal PrEP uptake and/or retention in those groups 48, 49.

The opportunity to investigate couples’ direct experiences with PrEP through multiple interviews conducted both jointly and individually is an important strength of this study, as they add to validity of the findings and the level of detail presented. However, we acknowledge that these data reflect the perspectives of Ugandan serodiscordant couples who had recently learned of and mutually disclosed their HIV serodiscordant status, who in most cases defined themselves as couples (rather than as having separated), and who were participating in a PrEP demonstration project. The experiences of couples who are not research participants, have long lived with serodiscordance, do not remain together, and/or whose nationalities and cultural backgrounds differ may not be the same. The similarities observed for Kenyan serodiscordant couples 23, 24, 25, 26 suggests the patterns described here are not characteristic only of Ugandans, however. Finally, the possibility that the qualitative interview data may be subject to social desirability bias, in which interviewees provide responses they believe to be “correct,” or “what the interviewer wants to hear,” must be acknowledged.

## 5 Conclusion

Because of its effectiveness in preventing HIV transmission, PrEP represented a solution to the problem of serodiscordance for Ugandan couples. Decreased fear during sex, renewed hopes for family building, and the prospect of eventual “live sex” were intermediate benefits serving this larger end. The most effective PrEP demand creation strategies may be those that meaningfully address the everyday life priorities of potential users, as well as HIV prevention. Understanding the meanings of PrEP for potential users can inform demand creation for PrEP scale‐up.

## Competing interests

The authors have no competing interests to declare.

## Authors’ contributions

NCW and MAW designed the qualitative research. ENJ and NCW analysed the data for this report, and wrote and revised the manuscript. MAW and EEP provided general supervision for the data collection process in Uganda, contributed to the data analytic process and reviewed and commented on drafts of the manuscript. TRM, ETK and SBA supervised data collection at the qualitative study sites. JMB and CLC provided feedback on emerging findings from the qualitative study and reviewed and provided comments on the manuscript. All authors critically reviewed and approved the final version.
